# Supplementary material for: Coexistence of Anaemia and Stunting among Children Aged 6–59 Months in Ethiopia: Findings from the Nationally Representative Cross-Sectional Study
Source: Int J Environ Res Public Health. 2023 Jun 29;20(13):6251. doi: 10.3390/ijerph20136251 (PMC10341109; doi:10.3390/ijerph20136251)
Supplement: Supplementary file 1 [file ijerph-20-06251-s001.zip › ijerph-2439528-supplementary.pdf]

Supplementary File S1: The flowchart for the sampling and data extraction procedure, Ethiopian Demographic and Health Survey (EDHS), 2005-2016

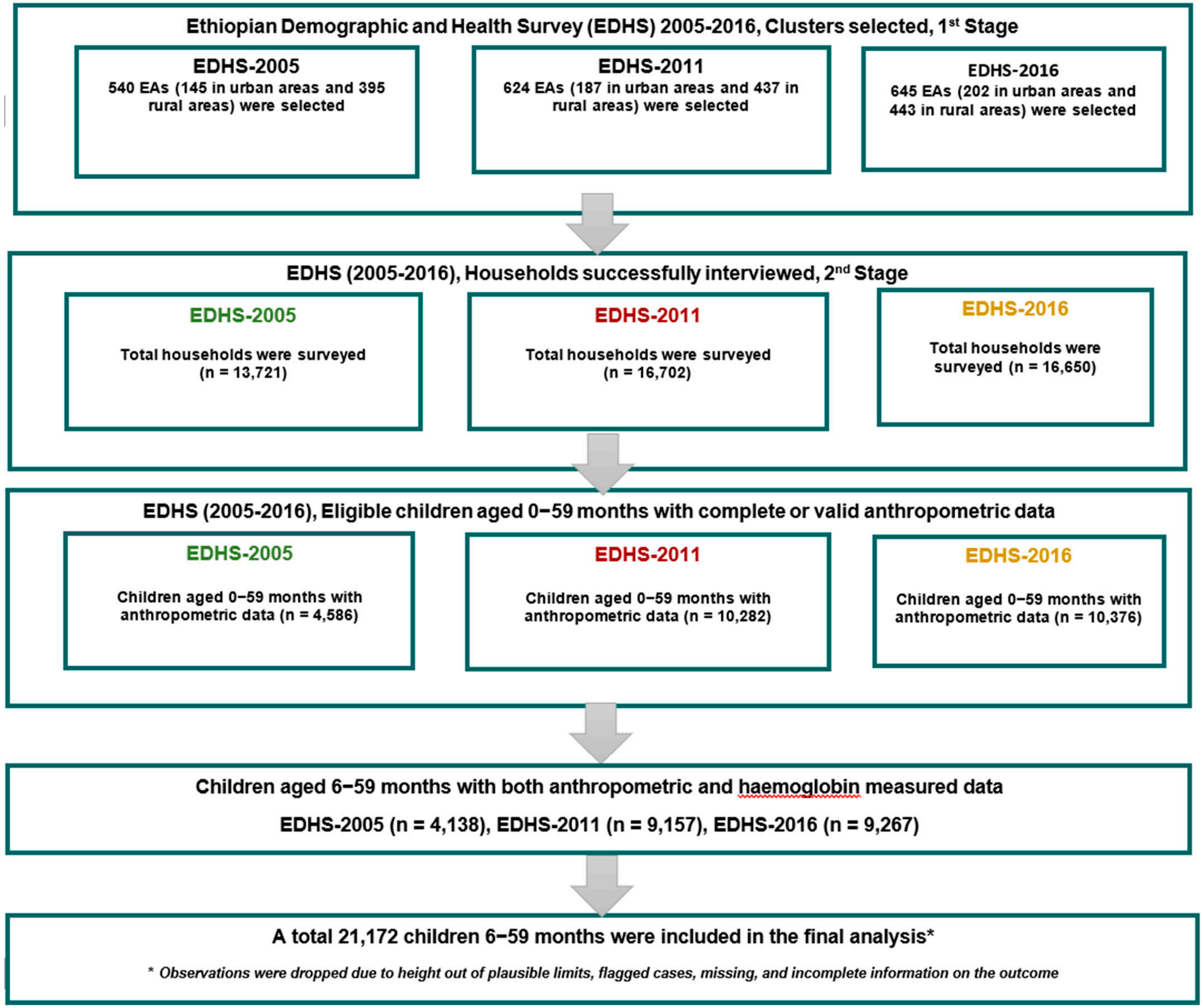

Supplementary File S2: A detailed list and variable coding of independent variables

**Lists of Variables**	**Categories**	**Descriptions**
**Individual level factors**		
Child factors		
Sex	1): Male, 2): Female	
Age of child (months)	1): 6-11, 2): 12-23, 3): 24-35, 4): 36-59	
Birth order	1): First born, 2): 2-4, 3): 5 or higher	
Birth interval	1): < 33 months, 2): ≥33 months	
Perceived size of child at birth	1): Larger , 2): Average, 3): Small	Assessed by the subjective reporting of the mother about the size of the child at birth and categorized as large, average and small. Birth size was used as a proxy measure of birth weight.
Currently breastfeeding	1): Yes, 2): No	
Received measles	1): Yes, 2): No	
Full vaccination	1): Yes, 2): No	"Yes" if the child received a Bacillus Calmette–Guérin (BCG) vaccination against tuberculosis; 3 doses of Diptheria, pertussis, and tetanus vaccine (DPT); ≥3 doses of polio vaccine (OPV); and 1 dose of measles vaccine; no otherwise.
Child had diarrhea in last 2 weeks	1): Yes, 2): No	
Child had fever in last 2 weeks	1): Yes, 2): No	
Child receive iron supplement	1): Yes, 2): No	Assessed by whether the child received iron supplement within the previous 7 days.
Received deworming medication in the last 6 month	1): Yes, 2): No	Assessed by whether the child received deworming table within the previous 6 months.
Parental factors		
Mother's age (years)	1): <18, 2): 18-24, 3): 25-34, 4): 35-49 years	
Mother's education status	1): No education, 2): Primary, 3): Secondary 4): Higher	
Mother's occupation	1): Not working, 2): Non agriculture, 3): Agriculture	
ANC Visit	1): None, 2): 1-3, 3): 4-7, 4): 8+	

Maternal BMI (kg/m^2)	1): <18.5 (underweight), 2): 18.5 to 24.9 (normal), 3): 25 +	
Maternal anemia	1): Yes, 2): No	
Maternal stature	1): Very short (<145 cm), 2): Short (145 to <155 cm), 3): Normal/Tall (155 to <200 cm)	
Listening to radio	1): Not at all, 2): Yes	
Watching television	1): Not at all, 2): Yes	
Household factors		
Household wealth category	1): Poor, 2): Middle, 3): Rich	The household wealth index was calculated based on household assets, such as televisions and bicycles. Principal components analysis was applied to generate the wealth index as a continuous scale of relative wealth. The wealth index was categorized into five wealth quintiles: 'very poor', 'poor', 'middle', 'rich' and 'very rich. For this analysis, we re-coded the wealth index into three categories for adequate sampling in each category: 'poor' (poor and very poor), 'middle' and 'rich' (rich and very rich).
Household Size	1): 1-4, 2): ≥ 5	
Place of cooking	1): In the house, 2): In separate building, 3): Outdoors	
Type of cooking fuel	1): Clean fuels, 2): Solid fuels	The type of cooking fuel were considered to be solid fuels if it is coal/lignite, charcoal, wood, straw/shrub/grass, crops, and animal dung. Categories such as electricity, liquefied petroleum gas (LPG), natural gas, and biogas were combined and coded as "no solid/ clean fuel use".
Toilet facility	1): Improved , 2): Unimproved, 3: Open defecation	Based on the WHO definition, facilities would be considered improved if any of the following occured: flush/pour flush toilets to piped sewer systems, septic tanks, and pit latrines; ventilated improved pit (VIP) latrines; pit latrines with slabs; and composting toilets. Unimproved sanitation included: flush or pour-flush to elsewhere; pit latrine without a slab or open pit; bucket, hanging toilet or hanging latrine. Other facilities,

		including households with no facility or use of bush/field, were considered as open defecation.
Source of drinking water	1): Improved , 2): Unimproved,	Improved sources of drinking water included piped water, public taps, standpipes, tube wells, boreholes, protected dug wells and springs, and rainwater. Other sources of drinking water were regarded as unimproved.
Household flooring	1): Improved , 2): Unimproved,	Household floors were considered to be unimproved if it is natural floors (earth/sand, dung), whereas rudimentary floors (wood planks, palm/bamboo), and finished floors (parquet or polished wood, vinyl or asphalt strips/plastic tile, ceramic tiles, cement, carpet) were considered as improved.
Time to get a water source	1): On premise, 2): ≤ 30 min, 3): 31-60 min, 4): >60 min	
Community Level Factors		
Residence	1): Urban, 2): Rural	
Region	1): Large centrals, 2): Small peripherals, 3): Metropolis	The geographical region of Ethiopia where household heads live. Tigray, Amhara, Oromia, and Sothern Nations Nationalities and Peoples Region (SNNPRs) were categorized under larger central regions; Afar, Somali, Benishangul, and Gambella were under Small peripherals, while Metropolis include Harari, Dire Dawa, and Addis Ababa regions
Ecological Zone	1): Tropical zone, 2): Subtropical zone, 3): Cool zone	Dry lowland (Kolla)- is below 1500 metres in elevation; Subtropical zone (Dry to Wet Cool Highlands) - includes the highlands areas of 1500 - 2500 metres in elevation (Weyna-Dega); Cold highland zone (Dega)- is above 2500 metres in elevation.
